# Zone-doubled Fresnel zone plates for high-resolution hard X-ray full-field transmission microscopy

**DOI:** 10.1107/S0909049512029640

**Published:** 2012-07-28

**Authors:** Joan Vila-Comamala, Yongsheng Pan, Jeffrey J. Lombardo, William M. Harris, Wilson K. S. Chiu, Christian David, Yuxin Wang

**Affiliations:** a Paul Scherrer Institut, 5232 Villigen PSI, Switzerland; b Advanced Photon Source, Argonne National Laboratory, Lemont, IL 60439, USA; c Department of Mechanical Engineering, University of Connecticut, Storrs, CT 06269, USA

**Keywords:** Fresnel zone plate, hard X-rays, full-field transmission X-ray microscopy, solid-oxide fuel cell

## Abstract

Full-field transmission X-ray microscopy is a unique non-destructive technique for three-dimensional imaging of specimens at the nanometer scale. Here, the use of zone-doubled Fresnel zone plates to achieve a spatial resolution better than 20 nm in the hard X-ray regime (8–10 keV) is reported. By obtaining a tomographic reconstruction of a Ni/YSZ solid-oxide fuel cell, the feasibility of performing three-dimensional imaging of scientifically relevant samples using such high-spatial-resolution Fresnel zone plates is demonstrated.

## Abstract 2

The use of zone-doubled Fresnel zone plates for sub-20 nm spatial resolution in full-field transmission X-ray microscopy and tomography at the hard X-ray regime (8–10 keV) is demonstrated.

## 1. Introduction

Full-field transmission X-ray microscopy (TXM) (Niemann *et al.*, 1976 ▶; Howells *et al.*, 2008 ▶; Sakdinawat & Attwood, 2010 ▶; Wang, 2012 ▶) is a powerful two- and three-dimensional imaging technique for the investigation of biological (Larabell & Nugent, 2010 ▶; Schneider *et al.*, 2010 ▶; Stampanoni *et al.*, 2010 ▶; Andrews *et al.*, 2010 ▶; Mokso *et al.*, 2012 ▶; Chichón *et al.*, 2012 ▶) and inorganic (Zschech *et al.*, 2008 ▶; Nelson *et al.*, 2011*a* ▶, 2012*a* ▶; Wang *et al.*, 2012 ▶; Liu *et al.*, 2012 ▶) materials at the nanometer scale. To date, transmission X-ray microscopy using state-of-the-art Fresnel zone plates (FZPs) (Attwood, 2000 ▶) as objective or focusing lenses has demonstrated a spatial resolution of about 10 nm in the soft sub-keV X-ray regime (Chao *et al.*, 2009 ▶; Vila-Comamala *et al.*, 2009 ▶; Rehbein *et al.*, 2012 ▶). Nevertheless, the use of the multi-keV X-ray regime for higher penetration power is often required to study inorganic materials and *in situ* applications. For hard X-ray energies (>8 keV) a spatial resolution below 30 nm has only recently been achieved (Chen *et al.*, 2008 ▶, 2011*a* ▶,*b* ▶) owing to the technological challenges involved in the production of high-aspect-ratio nanostructures. In FZP-based X-ray microscopes, the spatial resolution is limited by the width of the outermost zone of the FZP, Δ*r*, whereas the height of the diffractive structures has to be thick enough to provide a sufficient diffraction efficiency; that is the ratio between the amount of photons that are actually used by the lens to form the image or focal spot and the incoming X-ray intensity. To fulfill both requirements of high spatial resolution and adequate diffraction efficiency the fabrication of high aspect ratio is essential.

Here, we have used zone-doubled FZPs (Jefimovs *et al.*, 2007 ▶; Vila-Comamala *et al.*, 2011*b* ▶) with 20 nm outermost zone width to achieve hard X-ray full-field transmission microscopy with a spatial resolution better than 20 nm. The zone-doubling technique allows for the fabrication of high-aspect-ratio structures necessary for hard X-ray energies. The TXM set-up was then used to investigate the submicrometer structure of a nickel/yttrium-stabilized zirconia (Ni/YSZ) anode in a solid-oxide fuel cell (SOFC) (Izzo *et al.*, 2008 ▶; Shearing *et al.*, 2010*a* ▶,*b* ▶; Guan *et al.*, 2010 ▶; Grew *et al.*, 2010 ▶; Nelson *et al.*, 2011*a* ▶,*b* ▶). The performance and degradation of SOFCs are closely related to the materials that are used (Singhal, 2003 ▶; Zhu & Deevi, 2003 ▶; Brandon *et al.*, 2003 ▶; Atkinson *et al.*, 2004 ▶; McIntosh & Gorte, 2004 ▶). Among the challenges faced by SOFC research is the determination of materials and structures suitable for providing the necessary stability and durability while not compromising performance. This is particularly true for attempts to stabilize SOFCs by lowering their operational temperature and to investigate alternative materials for the use of hydrocarbon fuels and fuel reformates. The performance degradation observed in present SOFCs owing to the coking and poisoning of electrocatalysts and redox, thermal and chemical instabilities are among the most difficult issues to address. The tomographic reconstruction of the Ni/YSZ SOFC anode obtained here is a first step towards high-resolution *in situ* characterization (Nelson *et al.*, 2012*b* ▶) of nanostructures in such devices and to understand how their structural properties relate to their performance and degradation processes.

## 2. Methods and experimental set-up

The experiments were carried out using the full-field transmission X-ray microscope operating at the 32-ID beamline (Shen *et al.*, 2007 ▶) at the Advance Photon Source at Argonne National Laboratory. The X-ray illumination was provided by a 3.3 cm-period undulator source. A Si(111) double-crystal monochromator selected a monochromatic X-ray beam for the microscope. The estimated flux was 2 × 10^11^ photons s^−1^ at 8.0 keV.

The TXM optics were composed of a capillary condenser to provide illumination at the specimen plane; a 150 µm-diameter pinhole used in conjunction with a 250 µm beam stop upstream of the condenser lens to block the direct X-ray beam and produce a hollow cone illumination; and zone-doubled FZPs with an outermost zone width of 20 nm used as objective lens. The available capillary condenser provided a numerical aperture (NA) of approximately 1.9 mrad, equivalent to the NA of a FZP of 40 nm outermost zone width operating at 8.0 keV, while the 20 nm outermost zone width FZP had a NA of 3.7 mrad at the same energy. This led to a partially coherent illumination configuration that was not ideally suited to obtaining the optimum spatial resolution (Howells *et al.*, 2008 ▶; Born & Wolf, 1999 ▶). A detector system consisting of a single-crystal scintillator coupled to a CCD camera by a 10× visible-light objective lens was placed 1.7 m downstream of the FZP to record a magnified image of the sample. The magnification was 115× at the X-ray microscopy set-up, giving a total magnification of 1150×.

In the first part of the experiments we evaluated the spatial resolution by acquiring images at 9.0 and 10.0 keV photon energy of a spoked star test pattern made of gold with a smallest feature size of 30 nm. We used a zone-doubled FZP with a diameter of *D* = 100 µm. It was made of iridium and had a measured zone height of approximately 550 nm; that is, the structures had an aspect ratio above 25 at the outer regions of the lens. The expected working distances of the FZP range from 13.5 to 16.0 mm for X-ray energies from 8.0 to 10 keV. Such FZPs have demonstrated a diffraction efficiency of 3–5% at 8.0 keV (Vila-Comamala *et al.*, 2011*b* ▶). These diffraction efficiencies are roughly five times higher than those achieved by FZPs produced by more conventional fabrication techniques. Further details of its manufacture and characterization can be found by Vila-Comamala *et al.* (2010 ▶, 2011*a* ▶,*b* ▶).

In the second half of the experiment we studied a 10 µm-diameter cylinder extracted from a Ni/YSZ SOFC to assess the feasibility of performing tomographic imaging at high spatial resolution. The sample consisted of cermet material, that is, a composite material composed of one ceramic and one metallic phase. The 10 µm diameter suitable for the field of view of the TXM was prepared using a FEI Strata 400 DualBeam focused ion beam (FIB) system. The extraction was performed by first coating the bulk sample with a layer of gold and palladium to enhance the electrical conductivity of the surface. Next, the sample was loaded into the FIB system and a series of concentric circles were milled to eventually obtain a cylindrical formation inside the crater milled into the bulk material. Finally, the cylinder was attached to a computer-controlled microprobe by FIB-deposited platinum, detached from the bulk sample and transferred to the tip of a watch pin, so that the sample could be easily mounted into the full-field TXM set-up. A few particles of gold were manually dropped on the top of the cylinder in order to facilitate the alignment of the consecutive X-ray projection images obtained at different angles during the tomographic reconstruction process. In this part of the experiment a zone-doubled FZP with a diameter of *D* = 150 µm was used. A larger lens diameter was beneficial to provide a slightly larger working distance of about 20 mm at an X-ray energy of 8.35 keV which facilitated the accommodation of the specimen under study as well as a larger field of view. The photon energy was selected slightly above the nickel absorption edge to achieve optimal contrast of the nickel particles with respect to other components.

For the tomographic data acquisition, a total of 721 projections were recorded covering angles from 0 to 180°. Each image was acquired using an exposure time of 6 s resulting in a total time for recording the whole set of projections of about 75 min. The projections were used to compute the sample three-dimensional structure using the gridrec method (Dowd *et al.*, 1999 ▶), which consists of a gridding method with higher accuracy and efficiency than the filtered back-projection approach. The gridrec method achieves high performance because of its utilization of the prolate spheroidal wave function (Slepian & Pollack, 1961 ▶) for interpolation on the Cartesian grid. Using GPU-based computation, it took about 30 min to reconstruct an object three-dimensional image of size 2048^3^ voxels.

## 3. Results and discussion

**Figure 1:**
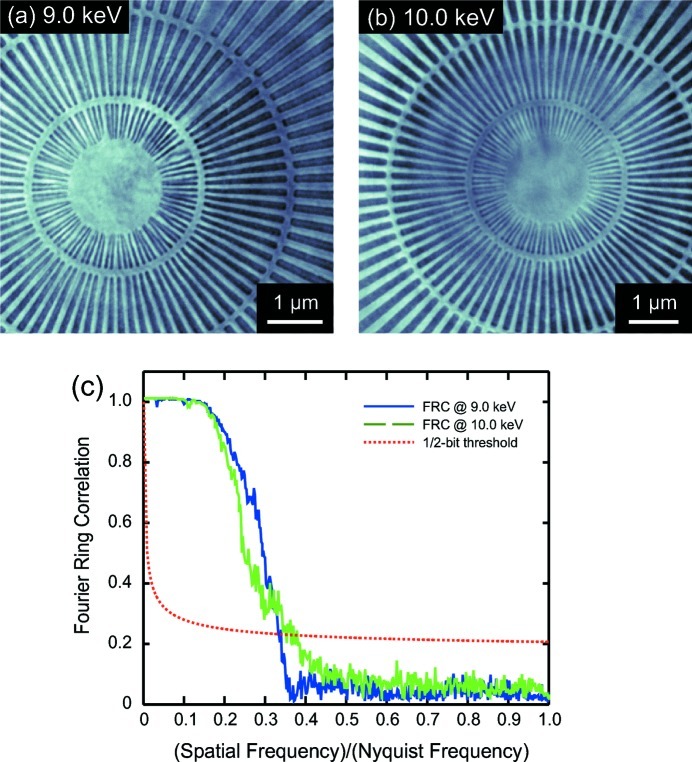
Full-field TXM images of the central region of a spoked star test pattern acquired using a zone-doubled Fresnel zone plate with an outermost zone width of 20 nm at (*a*) 9.0 keV and (*b*) 10.0 keV photon energy. The smallest pattern spokes of 30 nm width are clearly resolved. (*c*) Fourier ring correlation plot demonstrating a spatial resolution of 17 nm in the TXM images when considering a 1/2-bit SNR threshold curve.

Figs. 1(*a*) and 1(*b*) ▶ show images of the spoked star pattern at 9.0 and 10.0 keV. The finest spokes of 30 nm width are resolved. These two images were obtained by averaging of 20 images of 5 s acquisition time each and after subpixel precision alignment using an efficient image registration method based on cross-correlation (Guizar-Sicairos *et al.*, 2008 ▶). The decrease in contrast for the 10.0 keV photon energy image is attributed both to lower diffraction efficiency of the FZP and to the lower intrinsic absorption of the test object at higher energies.

We evaluated the spatial resolution of the TXM from the spoke star test pattern images using a Fourier ring correlation (FRC) method (Saxton & Baumeister, 1982 ▶; van Heel & Schatz, 2005 ▶; Vila-Comamala *et al.*, 2011*a* ▶). The FRC approach, also known as Fourier shell correlation when extended to three-dimensional imaging, is a well established method for estimating the resolution of two- and three-dimensional images in transmission electron microscopy. The FRC method provides a curve of the normalized cross-correlation coefficient between two independently acquired images at a given ring in the reciprocal space, which is then compared with a threshold curve chosen for a given signal-to-noise ratio (SNR) constant in the Fourier domain (van Heel & Schatz, 2005 ▶). To evaluate the spatial resolution at both photon energies, the original set of 20 images was divided into two subsets, for which averages were taken as the two independently acquired images required to apply the FRC method. The resulting FRC plots from this analysis and the 1/2-bit SNR threshold curve are shown in Fig. 1(*c*) ▶ as a function of the spatial frequency over the highest possible sampling frequency, *i.e.* the Nyquist frequency, which corresponds to the inverse of the image pixel size for our experimental conditions. The 1/2-bit SNR threshold curve has been taken from the derivation of van Heel & Schatz (2005 ▶). Taking into account at which spatial frequency over Nyquist frequency values the FRCs cut the 1/2-bit threshold curve and effective image pixel sizes of 6.0 and 6.7 nm, respectively, at 9.0 and 10.0 keV, we estimate a spatial resolution of 17 nm at both X-ray energies by multiplying the inverse values derived from the FRC plots by the pixel size.

To further characterize the zone-doubled FZP performance, we estimated its overall focusing diffraction efficiency to be 4% at 9.0 keV and 3% at 10.0 keV. The values were obtained by measuring the intensity of the illumination and the intensity of photons collected by the FZP. This result is consistent with previous measurements using a scanning transmission X-ray microscopy configuration at the European Synchrotron Radiation Facility (Vila-Comamala *et al.*, 2011*b* ▶).

**Figure 2:**
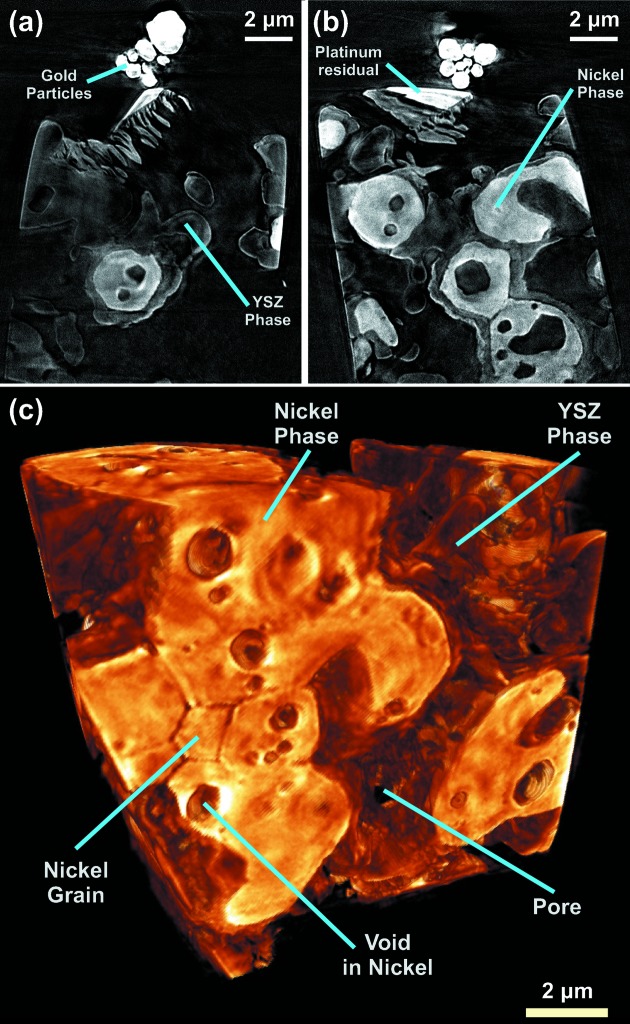
(*a*), (*b*) Slices and (*c*) rendering of the tomographic reconstruction of Ni/YSZ SOFC anode at 8.35 keV photon energy. The Ni and YSZ phases can be distinguished. The typical 120° dihedral angle of the nickel grain boundaries can be observed in (*c*).

Fig. 2 ▶ shows two slices, (*a*) and (*b*), and a three-dimensional rendering, (*c*), of the Ni/YSZ SOFC anode sample. In addition, two videos are available as supplementary material.^1^ The ceramic and metallic phases are easily distinguishable, with the brighter regions being nickel and the intermediate gray the yttrium-stabilized zirconia. Some platinum from the FIB milling of the sample and the gold particles used for alignment aid during the image processing are clearly located on top of the cylinder. In Fig. 2(*c*) ▶ one can observe the expected 120° dihedral angle of the nickel grain boundaries with thickness of about 40 to 60 nm. The improvement in the TXM spatial resolution enables more precise analysis of the Ni/YSZ structure. Knowledge of such characteristics as phase size distributions, phase contiguity, contact areas and the Ni/YSZ/pore triple phase boundary sites are important to the evaluation and prediction of the fuel cell performance. Because their structures and associated electrochemical processes inherently occur at small scales, improvements in imaging resolution are essential to understand and describe the behavior of the SOFC electrodes, as well as potentially other functional energy materials, at similar length scales (<100 nm).

## 4. Conclusion

We have demonstrated the use of zone-doubled diffractive X-ray lenses to achieve a spatial resolution of 17 nm on a test pattern by TXM in the hard X-ray regime. This resolution value was evaluated using the FRC method. It is significantly higher than what has been achieved by FZPs produced by more conventional fabrication methods and it approaches the spatial resolution scale in scanning electron microscopy in most practical applications. In addition, the high penetrating power of hard X-ray radiation allows for useful imaging through thick samples with no or minimum modification. The tomographic reconstruction of the 10 µm-diameter cylinder of the Ni/YSZ SOFC anode demonstrates the feasibility of performing three-dimensional imaging of relevant specimens using such high-spatial-resolution FZPs in the full-field TXM set-ups with prospects of *in situ* characterization (Nelson *et al.*, 2012*b* ▶). In future works, and by taking advantage of the flexibility of the TXM set-up, the interaction regions of devices such as batteries and fuel cells will be studied while in live operation. The specimens could be placed in extreme environments such as high temperature and or high pressure to investigate the material properties and synthesis processes. Even though the use of FZPs with 20 nm outermost zone width intrinsically shortens the working distance between the lens and sample and this fact could hinder the feasibility to accommodate complex sample environments, the zone-doubling technique can be directly employed to produce FZPs with larger diameters up to 1.0 mm (only restrained by the electron-beam lithography writing field) and regain the suitable longer working distances. By combining TXM imaging and high-resolution spectroscopy techniques such as differential absorption contrast and X-ray absorption near-edge spectroscopy (XANES) (Rau *et al.*, 2003 ▶; Meirer *et al.*, 2011 ▶; Nelson *et al.*, 2011*a* ▶), detailed elemental and chemical composition could also be dynamically tracked *in situ*. The current long times required for the acquisition of tomographic data will be shortened in future improvements, for example, by using faster detection cameras or developing FZPs with higher diffraction efficiency. High-resolution TXM will become a new valuable tool for studying mesoscale electrochemical reactions and developing new energy production and storage devices.

## Supplementary Material

Supplementary material file. DOI: 10.1107/S0909049512029640/pp5026sup1.mpg

Supplementary material file. DOI: 10.1107/S0909049512029640/pp5026sup2.mpg

## Footnotes

1 Supplementary data for this paper are available from the IUCr electronic archives (Reference: PP5026). Services for accessing these data are described at the back of the journal.
